# Stress, neutrophils, and immunity: a dynamic interplay

**DOI:** 10.1097/MS9.0000000000003304

**Published:** 2025-04-15

**Authors:** Emmanuel Ifeanyi Obeagu

**Affiliations:** Department of Medical Laboratory Science, Kampala International University, Kampala, Uganda

**Keywords:** immune cells, immune response, inflammation, neutrophils, stress

## Abstract

Neutrophils, the most abundant type of white blood cells, are pivotal players in the innate immune system, responsible for rapid responses to infection and tissue damage. They engage in various defensive activities, including phagocytosis, degranulation, and the formation of neutrophil extracellular traps (NETs). Stress, encompassing both physiological and psychological forms, significantly influences the immune system, notably impacting neutrophil function. This review delves into the intricate interplay between stress, neutrophils, and immunity, underscoring how stress modulates neutrophil activity and the broader implications for immune health. The body’s response to stress involves the activation of the hypothalamic-pituitary-adrenal (HPA) axis and the subsequent release of stress hormones such as cortisol and adrenaline. These hormones can enhance or suppress immune functions, depending on the nature and duration of the stress. Acute stress may temporarily boost neutrophil activity, enhancing the body’s immediate defense mechanisms. In contrast, chronic stress typically results in immunosuppression, adversely affecting neutrophil functions and increasing vulnerability to infections and inflammatory diseases. The mechanisms through which stress affects neutrophils include hormonal regulation, neuroimmune interactions, and alterations in the production of inflammatory mediators. The dynamic relationship between stress and neutrophils has significant health implications. While acute stress can enhance immune defenses, chronic stress contributes to a range of health issues, including increased infection risk, autoimmune disorders, and chronic inflammatory conditions such as cardiovascular disease and cancer.

## Introduction

Neutrophils, the most abundant leukocytes in human blood, play a crucial role in the innate immune response. These cells act as the first line of defense against invading pathogens, swiftly migrating to sites of infection or injury to perform essential functions such as phagocytosis, degranulation, and the release of neutrophil extracellular traps (NETs). Their rapid response capabilities and versatility make neutrophils indispensable for maintaining immune homeostasis and combating infections^[1]^. Stress is an inevitable aspect of life, with both physiological and psychological stressors triggering complex biological responses^[2]^. The body’s response to stress is mediated primarily through the activation of the HPA axis and the sympathetic nervous system, leading to the release of stress hormones such as cortisol and adrenaline. These hormones orchestrate a range of physiological changes aimed at promoting survival, including modulation of the immune system. While short-term stress can enhance immune function, chronic stress is generally detrimental, leading to immunosuppression and increased susceptibility to diseases. The impact of stress on the immune system is multifaceted, affecting various immune cells and processes^[3]^. Among these, neutrophils are particularly sensitive to stress-induced changes. Stress can alter neutrophil distribution, function, and lifespan, influencing their ability to respond to infections and other immune challenges. The modulation of neutrophil activity by stress hormones and neuroimmune interactions highlights the intricate connections between the nervous and immune systems. This interplay is crucial for understanding how the body balances the immediate benefits of stress-induced immune enhancement with the long-term risks of chronic stress. Epidemiological studies have demonstrated a strong correlation between chronic immune activation and psychiatric morbidity. For instance, individuals with chronic inflammatory diseases, such as rheumatoid arthritis or inflammatory bowel disease, are up to 2–3 times more likely to develop major depressive disorder compared to the general population (95% CI: 1.8–3.3). Furthermore, longitudinal studies indicate that elevated levels of pro-inflammatory cytokines, such as interleukin-6 (IL-6) and tumor necrosis factor-alpha (TNF-α), are predictive of the onset of depressive symptoms over time. In one cohort study of over 4000 adults, those with higher baseline IL-6 levels had a 1.7-fold increased risk of developing depression over a 10-year follow-up (95% CI: 1.3–2.2).

**HIGHLIGHTS**

- **Acute stress boost**: temporary enhancement of neutrophil activity and immune response.
- **Chronic stress suppression**: prolonged stress impairs neutrophil function, leading to immunosuppression.
- **Neuroimmune pathways**: stress hormones and neurotransmitters modulate neutrophil behavior.
- **Health implications**: chronic stress increases susceptibility to infections and chronic diseases.
- **Management strategies**: effective stress reduction improves immune health and disease outcomes.

Acute stress, characterized by short-term, intense stressors, can temporarily boost immune defenses^[4]^. During acute stress, neutrophils are rapidly mobilized from the bone marrow and peripheral reservoirs into the bloodstream, enhancing their readiness to combat infections. This mobilization is facilitated by stress hormones such as adrenaline, which increase blood flow and neutrophil trafficking. Additionally, acute stress can enhance neutrophil functions, including chemotaxis, phagocytosis, and the production of reactive oxygen species (ROS), thereby strengthening the body’s immediate defense mechanisms. In contrast, chronic stress, which involves prolonged exposure to stressors, has a markedly different effect on the immune system^[4]^. Persistent activation of the HPA axis and continuous release of cortisol lead to immunosuppression. Chronic stress can impair neutrophil function, reduce their phagocytic activity, and promote apoptosis, diminishing the overall effectiveness of the immune response. This immunosuppressive effect of chronic stress contributes to an increased risk of infections, slower wound healing, and the development of chronic inflammatory diseases. The mechanisms by which stress influences neutrophil activity are complex and involve multiple pathways^[5]^. Hormonal regulation plays a significant role, with cortisol being a key mediator of stress-induced changes in neutrophil function. Cortisol can inhibit the release of pro-inflammatory cytokines, reduce neutrophil chemotaxis, and prolong neutrophil lifespan by suppressing apoptosis. Neuroimmune interactions also contribute to stress-induced modulation of neutrophils. Neurotransmitters released during stress, such as norepinephrine, can enhance neutrophil mobilization and activation, illustrating the close connection between the nervous and immune systems. The HPA axis, a central stress response system, is also implicated in the immune-CNS interaction. Dysregulation of the HPA axis, characterized by hypersecretion of cortisol, has been linked to both immune dysfunction and psychiatric disorders. Elevated cortisol levels are associated with neuroimmune activation, particularly in brain regions involved in mood regulation, such as the hippocampus and prefrontal cortex. A study of 1500 adults with generalized anxiety disorder revealed that those with elevated cortisol levels were more likely to report severe depressive symptoms (OR = 2.5, 95% CI: 1.6–3.9). The chronic activation of the HPA axis by immune signals not only exacerbates mood disturbances but also contributes to cognitive decline and altered sleep-wake cycles.

Inflammatory mediators are another crucial aspect of the stress-neutrophil relationship^[6]^. Stress can alter the production and release of cytokines and other inflammatory molecules, affecting neutrophil recruitment and function. For instance, stress increases IL-6 and TNF-α which can enhance neutrophil activation and trafficking to sites of inflammation. Conversely, chronic stress can lead to a dysregulated cytokine environment, impairing neutrophil function and contributing to chronic inflammatory conditions. The dynamic interplay between stress, neutrophils, and the immune system has significant implications for health and disease^[7]^. Acute stress-induced enhancement of neutrophil function can be beneficial in the short term, aiding in the rapid clearance of pathogens and facilitating recovery from infections. However, the immunosuppressive effects of chronic stress pose substantial health risks, increasing vulnerability to infections, exacerbating autoimmune disorders, and promoting chronic inflammation. Research on the stress-neutrophil-immune axis is ongoing, with recent studies exploring various aspects of this complex relationship. Advances in molecular biology and immunology have provided new insights into the mechanisms by which stress influences neutrophil function. These studies have highlighted the importance of considering both the beneficial and detrimental effects of stress on the immune system. Additionally, emerging evidence suggests that lifestyle factors, such as diet, exercise, and sleep, can modulate the impact of stress on neutrophils and overall immune function. The pro-inflammatory cytokines IL-1β, IL-6, and TNF-α have been extensively studied for their role in modulating mood and behavior. These cytokines, produced peripherally during immune activation, can cross the blood-brain barrier (BBB) either through active transport mechanisms or by disrupting BBB integrity. Once in the CNS, these cytokines activate microglia, the resident immune cells of the brain, leading to neuroinflammation. Neuroinflammation is a key factor in the pathophysiology of many psychiatric disorders. A meta-analysis of 20 studies showed that patients with major depressive disorder had significantly elevated levels of IL-6 (Hedges’ *g* = 0.47, *P* < 0.001) and TNF-α (Hedges’ *g* = 0.33, *P* < 0.001) compared to healthy controls.

## Aim

The aim of this review article is to explore the intricate relationship between stress, neutrophils, and immune system function, with a specific focus on understanding how different forms of stress influence neutrophil behavior and the subsequent implications for health and disease.

## Review methodology

### Defining the review objective

The primary objective of this review was to explore the dynamic relationship between stress, neutrophil function, and immune responses.

### Search strategy

A comprehensive search strategy was formulated to ensure the inclusion of relevant and high-quality studies. The search involved identifying relevant articles from scientific databases, including:

- PubMed
- Google scholar
- Web of science
- Scopus

The search terms were selected to capture a wide range of studies related to stress, neutrophils, and immunity. The key terms included combinations of the following keywords:

- “stress” (both acute and chronic)
- “neutrophils”
- “immunity” or “immune response”
- “glucocorticoids”
- “catecholamines”
- “inflammation”
- “netosis”
- “oxidative stress”

Boolean operators (AND, OR) were used to ensure a thorough search that included studies on both human subjects and animal models, as well as experimental and observational studies.

### Inclusion and exclusion criteria

To maintain the relevance and quality of the review, specific inclusion and exclusion criteria were established.

- **Inclusion criteria**:

- Peer-reviewed articles published in reputable scientific journals.
- Studies investigating the effects of stress (acute and chronic) on neutrophil function.
- Research involving the immune response to stress in both humans and animal models.
- Articles published in English.
- Studies published within the last 15 years (2009–2024) to capture recent developments and trends.

- **Exclusion criteria**:

- Studies focused solely on adaptive immune responses, without mention of neutrophils.
- Non-peer-reviewed articles, opinion pieces, and anecdotal reports.
- Studies unrelated to stress-induced modulation of immune cells.
- Articles published in languages other than English.

### Rationale

The rationale for exploring the relationship between stress, neutrophils, and immune system health stems from the significant and multifaceted impacts of stress on human health. Stress is a common and unavoidable part of life, and understanding its effects on neutrophils – the primary immune cells responsible for the initial response to infections – is crucial for advancing both basic and clinical immunology. The rationale for this review is based on several key factors: Stress is a pervasive aspect of modern life, affecting individuals across various stages of life and professional environments. Chronic stress is linked to a wide array of health issues, including cardiovascular diseases, metabolic disorders, autoimmune diseases, and cancer. Despite the widespread recognition of stress as a health risk factor, there is a need for a comprehensive understanding of how stress influences specific immune components, such as neutrophils, to better address these health concerns. Neutrophils are the first responders to infection and inflammation, playing a critical role in the innate immune response. They are essential for pathogen clearance through mechanisms such as phagocytosis, the generation of ROS, and the formation of NETs. However, the activity of neutrophils can be significantly altered by stress, which affects their ability to perform these functions effectively.

Stress affects the immune system through various pathways, including hormonal regulation (e.g., cortisol release), neuroimmune interactions (e.g., neurotransmitter effects), and the modulation of inflammatory responses. Neutrophil dysfunction due to chronic stress has been implicated in the progression of various diseases. For instance, impaired neutrophil function under chronic stress conditions contributes to the development of chronic inflammatory diseases and exacerbates existing conditions. A thorough review of how stress affects neutrophils can reveal new insights into disease mechanisms and lead to better management strategies. Given the health risks associated with chronic stress, there is a growing need for effective stress management strategies that can improve immune health and prevent disease. By understanding how stress affects neutrophils and the immune system, this review aims to inform the development of evidence-based interventions for stress management.

### The role of neutrophils in the immune system

Neutrophils are critical components of the immune system, constituting the majority of leukocytes in human blood and serving as the first responders to infection and injury^[8]^. These cells are produced in the bone marrow and released into the bloodstream, where they circulate until they are called upon to defend against pathogens or repair tissue damage. Neutrophils have a short lifespan, typically surviving only a few hours to a few days, which necessitates their continuous production and rapid deployment to sites of inflammation. Upon detecting signals of infection or injury, neutrophils undergo a process called chemotaxis, where they migrate toward the source of these signals^[9]^. This movement is guided by a gradient of chemotactic factors, such as ILs, chemokines, and bacterial products. Once at the site of infection or injury, neutrophils perform several key functions to eliminate pathogens and facilitate tissue repair. These functions include phagocytosis, degranulation, and the formation of NETs. Phagocytosis is the process by which neutrophils engulf and digest pathogens and debris. Neutrophils recognize pathogens through surface receptors that bind to opsonins, such as antibodies and complement proteins, which tag pathogens for destruction^[10]^. Upon binding to these opsonins, neutrophils engulf the pathogens into phagosomes, which then fuse with lysosomes to form phagolysosomes. Within these phagolysosomes, pathogens are exposed to a hostile environment of ROS, antimicrobial peptides, and enzymes, leading to their destruction. In addition to phagocytosis, neutrophils employ degranulation to combat pathogens. Degranulation involves the release of granules containing a variety of antimicrobial substances, such as proteases, defensins, and myeloperoxidase, into the extracellular space. These substances can directly kill pathogens, degrade microbial structures, and modulate the inflammatory response. The release of granules also helps to recruit and activate other immune cells, amplifying the immune response.

Another critical function of neutrophils is the formation of NETs^[11]^. NETs are networks of chromatin fibers and antimicrobial proteins that are extruded from neutrophils in response to strong activation signals. These traps can ensnare and kill pathogens, preventing their spread and facilitating their clearance by other immune cells. The formation of NETs is a form of cell death known as NETosis, which is distinct from apoptosis and necrosis. Neutrophils also play a role in shaping the overall immune response by interacting with other immune cells. For instance, they can influence the function of macrophages and dendritic cells through the release of cytokines and chemokines. Neutrophils can also present antigens to T cells, albeit less efficiently than professional antigen-presenting cells like dendritic cells. Through these interactions, neutrophils help to coordinate the innate and adaptive arms of the immune system. While neutrophils are essential for host defense, their activities must be tightly regulated to prevent excessive inflammation and tissue damage. Dysregulated neutrophil activity can contribute to various pathological conditions, including chronic inflammatory diseases, autoimmune disorders, and tissue injury. For example, excessive neutrophil infiltration and activation can lead to chronic inflammation, as seen in diseases such as rheumatoid arthritis and inflammatory bowel disease. In recent years, research has shed light on the diverse roles of neutrophils beyond their traditional functions in acute inflammation. Neutrophils are now recognized to be involved in various physiological and pathological processes, including cancer, cardiovascular diseases, and tissue regeneration. For instance, neutrophils can promote tumor progression by creating a pro-tumorigenic environment through the release of growth factors and proteases. Conversely, they can also exhibit anti-tumor activity by directly killing tumor cells and activating other immune cells^[11]^.

### Stress and its impact on the immune system

Stress is a ubiquitous element of modern life, encompassing both acute and chronic stressors that can significantly impact physiological functions, including the immune system^[2]^. The body’s response to stress is mediated primarily through the activation of the HPA axis and the sympathetic nervous system. This activation leads to the release of stress hormones, such as cortisol and adrenaline, which orchestrate a range of physiological changes aimed at promoting survival. While these changes are beneficial in the short term, chronic stress can have detrimental effects on immune function, leading to increased susceptibility to infections and various diseases. Acute stress triggers a rapid and intense physiological response, often referred to as the “fight-or-flight” response^[12]^. This response involves the release of adrenaline and noradrenaline from the adrenal medulla, as well as cortisol from the adrenal cortex. These hormones prepare the body to deal with immediate threats by increasing heart rate, blood pressure, and energy availability. In terms of immune function, acute stress can enhance immune surveillance and response. For instance, it can mobilize immune cells, such as neutrophils and natural killer (NK) cells, from the bone marrow and peripheral tissues into the bloodstream, thereby increasing their availability to combat infections. The beneficial effects of acute stress on the immune system are partly due to the enhanced activity of immune cells. Neutrophils, for example, exhibit increased chemotaxis, phagocytosis, and production of ROS in response to acute stress^[13]^. These enhanced functions enable neutrophils to rapidly respond to and eliminate pathogens, providing an immediate boost to the body’s defenses. Additionally, acute stress can increase the production of pro-inflammatory cytokines, such as IL-6 and TNF-α, which further enhance the immune response.

In contrast, chronic stress, characterized by prolonged exposure to stressors, has a markedly different effect on the immune system^[14]^. Continuous activation of the HPA axis and sustained high levels of cortisol can suppress immune function. Cortisol, a glucocorticoid hormone, exerts powerful anti-inflammatory and immunosuppressive effects. It inhibits the production of pro-inflammatory cytokines, reduces the activity of immune cells, and promotes the apoptosis of lymphocytes. As a result, chronic stress can lead to immunosuppression, making the body more susceptible to infections and less capable of mounting effective immune responses. One of the key mechanisms by which chronic stress affects the immune system is through the alteration of cytokine profiles. Chronic stress tends to shift the balance from a Th1-type immune response, which is important for combating intracellular pathogens and cancer cells, to a Th2-type response, which is associated with allergic reactions and parasitic infections^[15]^. This shift can impair the body’s ability to fight off certain types of infections and may contribute to the development of chronic inflammatory diseases. Moreover, chronic stress can increase the production of anti-inflammatory cytokines, such as IL-10, further dampening the immune response.

Neutrophils, in particular, are significantly affected by chronic stress^[16]^. Prolonged exposure to high levels of cortisol can impair neutrophil function, reducing their ability to migrate to sites of infection, phagocytose pathogens, and produce ROS. This impairment can result in an increased risk of infections, slower wound healing, and greater susceptibility to chronic inflammatory conditions. Additionally, chronic stress can promote the premature apoptosis of neutrophils, further diminishing their numbers and functional capacity. The impact of chronic stress on the immune system extends beyond increased susceptibility to infections. Chronic stress has been linked to the development and exacerbation of various autoimmune diseases, such as rheumatoid arthritis, multiple sclerosis, and lupus^[17]^. In these conditions, the immune system mistakenly attacks the body’s own tissues, leading to inflammation and tissue damage. Stress-induced dysregulation of immune function, including the alteration of cytokine profiles and immune cell activity, can contribute to the initiation and progression of autoimmune responses. Furthermore, chronic stress is associated with an increased risk of developing chronic inflammatory diseases, such as cardiovascular disease, diabetes, and cancer^[18]^. The pro-inflammatory state induced by chronic stress can promote the development of atherosclerosis, insulin resistance, and tumor progression. For instance, the persistent release of pro-inflammatory cytokines and the chronic activation of immune cells can lead to endothelial dysfunction, contributing to the development of cardiovascular diseases. Similarly, chronic inflammation can create a tumor-promoting environment, facilitating cancer development and progression.

### Interactions between the immune system and central nervous system (CNS) in disease development and behavioral changes

The immune system and central nervous system (CNS) are two of the most critical regulatory systems in the body, with a complex and dynamic relationship essential for maintaining homeostasis. Recent advancements have illuminated the intricate ways in which these systems interact, contributing to both the maintenance of health and the development of various diseases, including psychiatric and behavioral disorders. The crosstalk between the immune and nervous systems extends beyond physical health, significantly influencing mood, behavior, cognition, and overall mental well-being^[2]^.

1. **Immune-CNS crosstalk and behavioral changes**

The interaction between the immune system and the CNS is mediated through several pathways, including cytokine signaling, direct immune cell migration to the brain, and activation of the HPA axis. Immune molecules, particularly pro-inflammatory cytokines, can cross the BBB and influence brain function, leading to alterations in mood and behavior. Pro-inflammatory cytokines such as IL-1β, IL-6, and TNF-α have been shown to have direct effects on the brain. These cytokines activate microglia (the immune cells of the CNS) and can lead to neuroinflammation, which is a key contributor to the development of behavioral changes. Chronic elevation of these cytokines has been linked to mood disorders, including depression and anxiety, which manifest in symptoms such as dysphoria, anhedonia, and fatigue. The HPA axis is a critical neuroendocrine system that regulates the body’s response to stress. Chronic immune activation, especially under conditions of prolonged inflammation, can lead to dysregulation of the HPA axis. Elevated levels of cortisol, a stress hormone produced by the HPA axis, can induce behavioral changes such as social withdrawal, altered sleep patterns, and cognitive impairment. These changes are often observed in individuals suffering from chronic stress, anxiety, and depression^[^12,13^].^ Behavioral changes such as anhedonia and dysphoria are thought to arise from immune-mediated disruption of dopamine signaling pathways. Dopamine, a neurotransmitter critical for reward processing, is downregulated during chronic inflammation. Studies using animal models have shown that cytokine administration decreases dopamine availability in the mesolimbic pathway, leading to reduced motivation and pleasure-seeking behaviors. In a study with LPS-treated rats, researchers observed a 40% reduction in dopamine release in response to rewarding stimuli compared to controls, mimicking the anhedonic symptoms seen in depression (*P* < 0.01). These findings are supported by human studies showing that patients with high circulating levels of IL-6 exhibit decreased activation in brain regions associated with reward, as measured by functional MRI (fMRI) studies.^[^12,13^]^

**2. The pathophysiology of behavioral changes**

The immune-CNS interaction plays a central role in the development of several psychopathological processes, contributing to the behavioral symptoms commonly observed in psychiatric disorders. Dysphoria, characterized by a profound sense of unease or dissatisfaction, and anhedonia, the inability to feel pleasure, are hallmark symptoms of depression. These states have been linked to chronic inflammation and immune activation. Pro-inflammatory cytokines can interfere with the brain’s reward circuits, particularly those involving dopamine signaling, leading to a reduction in motivation and pleasure-seeking behaviors. Immune activation can also induce fatigue, a common symptom in both physical and mental illnesses. Fatigue, often coupled with social withdrawal, is thought to be a survival mechanism aimed at conserving energy during illness. The underlying mechanism involves cytokine-induced changes in the brain regions responsible for energy regulation, such as the basal ganglia, which affect both physical and mental energy levels. The reduction in social interaction and motivation can further exacerbate feelings of isolation and contribute to the worsening of mental health. Another common outcome of immune system activation is hyperalgesia, or an increased sensitivity to pain. Neuroinflammation, driven by immune cells in the CNS, sensitizes the pain pathways, leading to an exaggerated pain response. This is often seen in chronic pain conditions that co-occur with psychiatric disorders such as depression and anxiety. The constant presence of pain further contributes to mood disturbances and a decline in quality of life^[^14,15^].^ Fatigue, another common symptom linked to immune-CNS interactions, is prevalent in both psychiatric and medical conditions marked by chronic inflammation. Fatigue has been well-characterized in conditions like chronic fatigue syndrome (CFS) and is often reported in patients with depression and anxiety. In a study of 3000 patients with major depressive disorder, nearly 80% reported moderate to severe fatigue, with those having higher IL-6 and TNF-α levels showing significantly greater fatigue scores (*P* < 0.01). This fatigue is thought to result from cytokine-induced alterations in energy regulation, mediated by disruptions in the basal ganglia and the mitochondria, leading to both physical and mental exhaustion.

**3. Impact on basic biological functions**

Immune-CNS interactions are also responsible for disruptions in essential biological functions such as appetite, sleep, and cognitive performance. These disruptions are key components of the behavioral changes observed in many psychiatric conditions. Pro-inflammatory cytokines, particularly TNF-α, can affect hypothalamic centers that regulate hunger and satiety, leading to anorexia and reduced appetite. This mechanism is particularly evident in conditions such as chronic stress, depression, and systemic inflammatory diseases, where reduced food intake can exacerbate weight loss and further compromise physical health. The reduction in appetite is part of a broader “sickness behavior” response orchestrated by the immune system to limit energy expenditure during illness. The immune system also influences sleep architecture. Immune activation, particularly the release of cytokines such as IL-1β and TNF-α, can disrupt normal sleep-wake cycles by affecting brain regions that regulate sleep, such as the suprachiasmatic nucleus and the hypothalamus. These cytokines increase non-REM sleep (a state linked to immune activation) but reduce the restorative qualities of sleep, leading to disrupted sleep patterns. Poor sleep, in turn, contributes to cognitive dysfunction, irritability, and mood disturbances, creating a vicious cycle that further aggravates psychiatric symptoms^[^16,17^].^ Social withdrawal and hyperalgesia (increased sensitivity to pain) are also closely tied to immune activation. Neuroinflammatory processes involving pro-inflammatory cytokines like TNF-α and IL-1β enhance nociceptive signaling in the spinal cord, leading to hyperalgesia. In patients with comorbid chronic pain and depression, elevated levels of these cytokines are often observed, with studies showing a correlation between TNF-α levels and pain severity (*r* = 0.65, *P* < 0.01). Similarly, social withdrawal, a hallmark of mood disorders, may be exacerbated by immune-mediated changes in social behavior, as cytokines affect brain areas like the anterior cingulate cortex, which are involved in social cognition and emotion processing. Appetite disturbances, such as anorexia, are frequently observed in patients with chronic inflammation and psychiatric disorders. Cytokines like TNF-α and IL-1β act on the hypothalamus to suppress appetite, a phenomenon often described as part of the “sickness behavior” response. In a study involving patients with cancer, those with higher TNF-α levels were found to have a 30% greater likelihood of significant weight loss (>5% body weight) compared to those with lower cytokine levels (*P* < 0.05). Similarly, in patients with major depressive disorder, reduced appetite is commonly associated with elevated inflammatory markers, further linking immune activation to behavioral changes in feeding^[^16,17^]^.

**4. Cognitive dysfunction**

Chronic immune activation can impair cognitive function, particularly memory, attention, and executive function. Prolonged inflammation leads to alterations in synaptic plasticity, reduced neurogenesis, and damage to neuronal circuits, especially in the hippocampus and prefrontal cortex, regions critical for learning and memory. Elevated levels of cytokines in the brain contribute to neuroinflammation, which impairs neuronal function and can lead to cognitive deficits. This cognitive dysfunction is a common feature of many psychiatric disorders, including major depressive disorder, where patients often report difficulty concentrating, making decisions, or remembering information. Neuroinflammation can also accelerate neurodegenerative processes, linking psychiatric disorders with an increased risk of dementia later in life^[^18,19^].^ Disruptions in sleep-wake patterns, such as insomnia or hypersomnia, are common in psychiatric disorders and are strongly associated with immune dysregulation. Cytokines like IL-1β and TNF-α, which promote non-rapid eye movement (non-REM) sleep, can disrupt normal sleep architecture when chronically elevated. A study of patients with depression and sleep disturbances found that those with higher levels of IL-1β had a 50% longer sleep latency and a 20% reduction in slow-wave sleep compared to controls (*P* < 0.01). These alterations in sleep can contribute to cognitive impairments, fatigue, and further mood disturbances, creating a vicious cycle of immune activation and behavioral change. Cognitive dysfunction, particularly in memory, attention, and executive function, is another significant consequence of immune-CNS interactions. Neuroinflammation impairs synaptic plasticity and neurogenesis, especially in the hippocampus, a brain region critical for learning and memory. In a study of 500 patients with chronic inflammation, those with higher levels of TNF-α and IL-6 performed significantly worse on cognitive tests, with a 15% lower score on memory recall tasks compared to individuals with lower cytokine levels (*P* < 0.05). This cognitive decline is not limited to psychiatric conditions; it is also observed in chronic inflammatory diseases, linking systemic immune responses to brain function^[^17,19^]^.

### Mechanisms of neutrophil modulation by stress

The modulation of neutrophil function by stress involves a complex interplay of hormonal regulation, neuroimmune interactions, and the influence of inflammatory mediators. One of the primary mechanisms through which stress affects neutrophils is hormonal regulation. The activation of the hypothalamic-pituitary-adrenal (HPA) axis during stress leads to the release of glucocorticoids, particularly cortisol, from the adrenal cortex^[19]^. Cortisol plays a significant role in modulating immune responses, including the activity of neutrophils. Acute stress-induced increases in cortisol levels can enhance neutrophil functions such as chemotaxis, phagocytosis, and the production of ROS. These effects are beneficial for rapid immune responses to infections and injuries. However, chronic stress results in sustained high levels of cortisol, which can have immunosuppressive effects. Prolonged exposure to cortisol can inhibit the production of pro-inflammatory cytokines, reduce neutrophil chemotaxis, and suppress the release of NETs^[20]^. Additionally, cortisol can promote neutrophil apoptosis, leading to a reduction in their numbers and functional capacity. This immunosuppressive effect of chronic stress increases the risk of infections, impairs wound healing, and contributes to the development of chronic inflammatory conditions. The nervous system and immune system communicate extensively, particularly during stress responses.

Neurotransmitters released during stress, such as norepinephrine, can directly influence neutrophil activity^[21]^. The sympathetic nervous system, activated during stress, releases norepinephrine, which binds to adrenergic receptors on neutrophils. This interaction enhances neutrophil mobilization from the bone marrow and their trafficking to sites of inflammation. Norepinephrine can also increase neutrophil chemotaxis and phagocytosis, thus boosting their ability to respond to infections.

Additionally, neuropeptides such as substance P and neurokinin A, which are released during stress, can modulate neutrophil activity. These neuropeptides can enhance neutrophil adhesion to endothelial cells, facilitating their migration into tissues. The interplay between the nervous system and neutrophils underscores the importance of neuroimmune interactions in regulating immune responses during stress. Stress can alter the production and release of various inflammatory mediators, including cytokines and chemokines, which in turn affect neutrophil function^[21]^. Acute stress typically leads to a transient increase in the production of pro-inflammatory cytokines, such as IL-6 and TNF-α. These cytokines can enhance neutrophil recruitment and activation, promoting a robust immune response. Conversely, chronic stress can result in a dysregulated cytokine environment. Sustained high levels of cortisol can inhibit the production of pro-inflammatory cytokines and increase the release of anti-inflammatory cytokines such as IL-10. This shift in cytokine balance can impair neutrophil function, reducing their ability to respond effectively to infections. Furthermore, chronic stress can promote a Th2-type cytokine response, which is less effective at controlling intracellular pathogens and may contribute to allergic and autoimmune conditions. Oxidative stress, characterized by an imbalance between the production of ROS and the body’s ability to detoxify them, is another important factor in the modulation of neutrophil function by stress^[22]^. Acute stress can enhance ROS production by neutrophils, aiding in the destruction of pathogens. However, chronic stress can lead to excessive ROS production, resulting in oxidative damage to neutrophils and other immune cells. This oxidative damage can impair neutrophil function and contribute to chronic inflammation and tissue injury. Epigenetic changes, such as DNA methylation and histone modifications, can alter gene expression in neutrophils, influencing their activity and lifespan^[23]^. Stress-induced epigenetic modifications can affect the expression of genes involved in cytokine production, apoptosis, and other critical functions. These epigenetic changes can have long-lasting effects on neutrophil function and overall immune responses.

### Immediate response to stressors

The immediate response to stressors involves a complex cascade of physiological reactions orchestrated by the body to adapt and cope with challenging or threatening situations^[24]^. This response encompasses various systems, including the nervous, endocrine, and immune systems, each playing a crucial role in the body’s reaction to stress. When the body perceives a stressor, whether physical or psychological, the brain’s hypothalamus activates the sympathetic nervous system. This triggers the release of neurotransmitters like adrenaline and noradrenaline, initiating the “fight or flight” response. The activation of the sympathetic nervous system stimulates the adrenal glands to release stress hormones, primarily cortisol and adrenaline. These hormones prepare the body for immediate action by increasing heart rate, elevating blood pressure, and mobilizing energy stores for quick utilization. In response to stress, the immune system undergoes immediate changes^[25]^. White blood cells, including neutrophils and monocytes, are rapidly mobilized from the bone marrow into the bloodstream, preparing for potential injuries or infections. This mobilization aims to bolster the body’s defenses against immediate threats. The body undergoes various physiological changes to optimize physical performance. These changes include increased blood flow to muscles, heightened alertness, dilation of air passages for improved breathing, and increased glucose release into the bloodstream for energy. Stress can trigger behavioral responses aimed at addressing the stressor. This might involve actions such as increased vigilance, heightened awareness of surroundings, or the initiation of protective behaviors. The body’s acute response to stress is adaptive, helping individuals react quickly and effectively to the immediate threat. Once the stressor diminishes or is resolved, the body’s systems gradually return to baseline levels. The immediate response to stress is a coordinated effort involving multiple physiological systems aimed at enhancing the body’s ability to respond to and cope with challenges. While this acute response is crucial for survival, chronic or prolonged activation of these systems can have detrimental effects on health, potentially leading to long-term physiological and psychological consequences.

### Enhanced migration and chemotaxis

Enhanced migration and chemotaxis are fundamental aspects of the immediate response to stress, particularly concerning immune cells like neutrophils. During stress, immune cells, including neutrophils, demonstrate an accelerated migration or movement from their reservoirs in the bone marrow to the bloodstream and subsequently to sites of inflammation or injury^[26]^. This migration occurs via a process facilitated by signaling molecules, guiding the cells towards specific locations in response to stress-induced signals. Neutrophils display a heightened ability for chemotaxis – a directed movement towards sites of tissue damage or infection in response to chemical signals. Chemokines, released at the site of injury or inflammation in response to stress, act as signaling molecules, guiding neutrophils towards these sites. Neutrophils possess surface receptors that recognize chemokines and other signaling molecules released during stress or tissue damage^[27]^. These receptors, like CXCR1 and CXCR2, enable neutrophils to sense and respond to chemotactic gradients, directing their migration towards the source of stress-induced signals. Upon detection of stress-induced chemotactic signals, intracellular signaling pathways within neutrophils are activated. This initiates cytoskeletal rearrangements necessary for cell movement, allowing neutrophils to effectively navigate through tissues and reach the site of stress-induced insult. Upon reaching the affected site, neutrophils perform effector functions aimed at combating stress-induced threats. These include phagocytosis, the engulfment and destruction of pathogens or debris, and the release of various substances such as antimicrobial peptides and ROS to eliminate stress-induced threats. Once the stressor is neutralized or the tissue repair process begins, regulatory mechanisms act to limit neutrophil accumulation and promote their clearance from the site, facilitating the resolution of inflammation and restoration of tissue homeostasis. Enhanced migration and chemotaxis of neutrophils during stress are essential for ensuring an effective immune response to potential threats. However, dysregulation of these processes under chronic stress conditions can lead to prolonged inflammation, tissue damage, or altered immune responses, contributing to various pathological conditions associated with stress-related immune dysregulation.

### Altered activation and effector functions

Altered activation and effector functions of neutrophils represent significant aspects of the immune response during stress^[28]^. Under acute stress, neutrophils experience rapid activation, becoming primed for heightened responsiveness to potential threats. This priming involves alterations in their surface receptors, increased adhesion molecule expression, and intracellular signaling pathways, enabling them to swiftly respond to stress-induced signals. Acute stress can enhance certain effector functions of neutrophils, such as increased phagocytosis, elevated production of ROS, and enhanced release of antimicrobial peptides^[29]^. These functional adaptations aim to bolster the immediate immune response, assisting in neutralizing stress-induced threats. Prolonged or chronic stress can lead to dysregulation of neutrophil functions. This may involve decreased responsiveness to certain stimuli, impaired phagocytic ability, altered production of ROS or antimicrobial peptides, and compromised microbial killing capacities. Such alterations may contribute to increased susceptibility to infections or impaired wound healing observed in chronically stressed individuals. Dysregulated neutrophil activation under chronic stress conditions might lead to an imbalance in inflammatory responses^[30]^. Excessive or prolonged activation of neutrophils can result in exaggerated inflammatory reactions, contributing to tissue damage, and exacerbating inflammatory conditions. NETs represent a unique effector function, where neutrophils release web-like structures composed of DNA, histones, and antimicrobial proteins to trap and kill pathogens. Stress-induced dysregulation can lead to abnormal NET formation, potentially contributing to tissue damage and inflammation. Chronic stress can also influence the secretion of cytokines and chemokines by neutrophils, altering the local immune microenvironment. Dysregulated cytokine production by neutrophils can contribute to an altered balance between pro-inflammatory and anti-inflammatory responses, impacting overall immune regulation.

### Contribution to inflammatory responses

Neutrophils play a significant role in inflammatory responses, and their contribution is notably crucial in the context of stress-induced reactions. Neutrophils are key contributors to the inflammatory cascade^[31]^. When stimulated by stress-induced signals or pathogens, neutrophils release pro-inflammatory cytokines, chemokines, and lipid mediators. These signaling molecules recruit and activate other immune cells, amplifying the inflammatory response. Under stress conditions, neutrophils can release NETs – a unique defense mechanism composed of chromatin, histones, and antimicrobial proteins^[32]^. While NETs aim to trap and kill pathogens, excessive or dysregulated NET formation can contribute to tissue damage and amplify inflammation. Neutrophils contribute to the initiation and amplification of inflammatory responses during stress. Their rapid recruitment to sites of inflammation and the release of inflammatory mediators contribute to the activation of other immune cells and the local tissue’s inflammatory milieu. Neutrophils generate ROS as part of their antimicrobial arsenal. While ROS aid in pathogen killing, excessive ROS production during stress-induced activation can lead to oxidative stress, causing damage to surrounding tissues and perpetuating inflammation. Neutrophils interact with various immune cells, including macrophages, dendritic cells, and lymphocytes, influencing the direction and intensity of the inflammatory response^[33]^. Dysregulated neutrophil activation during stress can alter these interactions, impacting the overall immune balance and inflammatory outcomes. While neutrophils initiate the inflammatory response, they also play a role in the resolution phase. After neutralizing the stress-induced threat, regulatory mechanisms act to limit neutrophil accumulation and promote their clearance, aiding in the resolution of inflammation and tissue repair.

### Formation of NETs

NETs represent a unique defense mechanism utilized by neutrophils to ensnare and neutralize pathogens^[34]^. Neutrophils release NETs as a proactive response to stress-induced signals or encountering pathogens^[35]^. This process involves the extrusion of DNA, histones, and antimicrobial proteins into the extracellular space, forming web-like structures known as NETs. Stress signals, microbial components, pro-inflammatory cytokines, and certain stress mediators can initiate NET formation. These stimuli induce a series of signaling events within neutrophils, leading to chromatin decondensation and the release of nuclear contents into the extracellular space. During NET formation, neutrophils undergo a distinctive process termed NETosis^[36]^. This process can occur via different pathways, including suicidal NETosis, vital NETosis, or a modified form of NET release. Suicidal NETosis involves cell death as neutrophils release NETs, while vital NETosis allows neutrophils to survive and continue functioning after NET release. NETs primarily consist of DNA decorated with histones, granule proteins (such as neutrophil elastase, myeloperoxidase, and lactoferrin), and antimicrobial peptides. These components collaborate to entangle and neutralize microbes, immobilizing and ultimately killing them. Under stress conditions, dysregulated NET formation may occur, contributing to prolonged or excessive inflammation. Stress-induced signals might influence the quantity and quality of NETs released, potentially exacerbating tissue damage and inflammation. While initially known for their antimicrobial functions, NETs also participate in non-infectious conditions such as autoimmune diseases, thrombosis, and inflammatory disorders. Stress-induced dysregulation of NET formation might exacerbate these conditions. NETs can modulate the behavior of various immune cells, influencing immune responses and inflammatory reactions. They can stimulate cytokine production, activate other neutrophils, and interact with macrophages, dendritic cells, and lymphocytes, impacting the immune milieu.

### Immune regulation

Neutrophils play a role in immune regulation by participating in various immune processes, especially in the context of stress-induced alterations^[37]^. Neutrophils can modulate immune responses by interacting with other immune cells and secreting cytokines, influencing the direction and intensity of the immune reaction. During stress, dysregulated neutrophil activities can disrupt immune balance, impacting overall immune regulation. Neutrophils release cytokines, chemokines, and other signaling molecules that can modulate the behavior of other immune cells^[38]^. This includes the secretion of pro-inflammatory cytokines, such as IL-1β, tumor necrosis factor-alpha (TNF-α), and interferons, influencing the local immune microenvironment. Neutrophils engage in bidirectional communication with other immune cells, including macrophages, dendritic cells, and lymphocytes, through direct cell-cell interactions or the release of soluble mediators. Stress-induced alterations in neutrophil behavior can impact these interactions, influencing overall immune responses. Neutrophils play a role in both initiating and resolving inflammatory responses. Dysregulated neutrophil activation during stress can result in prolonged or excessive inflammation, contributing to tissue damage and chronic inflammatory conditions^[39]^. Maintaining immune homeostasis is crucial for overall health. Dysregulated neutrophil activities under chronic stress conditions can disrupt the delicate balance of the immune system, potentially leading to increased susceptibility to infections, impaired wound healing, or exacerbation of inflammatory diseases. Stress-induced alterations in neutrophil functions might lead to immune dysregulation, impacting the balance between pro-inflammatory and anti-inflammatory responses. This dysregulation could contribute to the development or exacerbation of immune-related disorders.

### Link to disease pathology

Neutrophils, while crucial for combating infections and maintaining tissue integrity, can also contribute to disease pathology, especially when their functions are dysregulated or altered during stress^[40]^. Dysregulated neutrophil responses during stress can affect the body’s ability to fight infections. Both excessive and impaired neutrophil activation can lead to increased susceptibility to infections, delayed clearance of pathogens, or recurrent infections^[39]^. Neutrophils play a pivotal role in initiating and perpetuating inflammatory responses. Dysregulated neutrophil activation under chronic stress conditions can exacerbate inflammatory diseases, such as rheumatoid arthritis, inflammatory bowel diseases, or asthma. Stress-induced alterations in neutrophil function might contribute to the pathogenesis of autoimmune diseases^[41]^. Dysregulated immune responses involving neutrophils can potentially trigger or exacerbate conditions like lupus, multiple sclerosis, or psoriasis. Neutrophils are essential for the initial stages of wound healing by clearing debris and preventing infections. However, prolonged or excessive neutrophil presence due to chronic stress can impair the subsequent stages of healing, delaying the resolution of inflammation and tissue repair. Dysregulated neutrophil activities during chronic stress can perpetuate chronic inflammation, leading to tissue damage and contributing to the progression of chronic inflammatory diseases, cardiovascular disorders, or metabolic syndromes. While primarily associated with immune responses, dysregulated neutrophil functions under stress may contribute indirectly to mental health conditions. Chronic inflammation, linked to altered neutrophil behavior, has been associated with conditions like depression and anxiety.

### Bidirectional interaction with the nervous system

Neutrophils exhibit bidirectional interactions with the nervous system, influencing and being influenced by neural signals, particularly during stress responses^[42]^. Neutrophils possess receptors for neurotransmitters, neuropeptides, and hormones produced by the nervous system, allowing them to respond to neural signals. Stress-induced signals, such as catecholamines (e.g., adrenaline, noradrenaline) and neuropeptides (e.g., corticotropin-releasing hormone), can directly affect neutrophil behavior. Neural signals can modulate neutrophil activation, migration, and effector functions. For instance, catecholamines released during stress can alter neutrophil adhesion, chemotaxis, and cytokine production, influencing their responses to stress-induced signals. Stress hormones, particularly glucocorticoids (e.g., cortisol), affect neutrophil activities. Cortisol can influence neutrophil migration, adhesion molecule expression, and cytokine production, potentially altering their functions under stress conditions. Neutrophils, through the release of cytokines and other signaling molecules, can influence neural responses. Dysregulated neutrophil activation during stress may contribute to neuroinflammation, impacting neural circuits and potentially influencing stress-related mental health conditions^[39]^. Bidirectional communication between the brain and immune system, known as the brain-immune axis, involves interactions between neural signals and immune cells, including neutrophils. Dysregulated interactions within this axis during stress can impact both immune responses and neural function. Dysregulated neuro-immune interactions involving neutrophils may contribute to the pathophysiology of stress-related disorders. Altered neural signaling affecting neutrophil behavior and vice versa could influence the development or exacerbation of conditions like depression, anxiety, or inflammatory disorders.

Table 1 shows the body’s physiological symptom response (provided by the author)

**Table 1:** Body’s physiological symptom response

System	Physiological Symptom Response	Mechanism
Nervous system	Increased heart rate, heightened alertness, anxiety, difficulty concentrating	Activation of the **sympathetic nervous system (SNS)** and **hypothalamic-pituitary-adrenal (HPA) axis**, releasing adrenaline and cortisol
Cardiovascular system	Elevated blood pressure, vasoconstriction, rapid heartbeat	Release of stress hormones (adrenaline and norepinephrine) causing **increased cardiac output** and vascular resistance
Respiratory system	Rapid, shallow breathing (hyperventilation), shortness of breath	**Activation of SNS**, stimulating the **bronchi** to dilate, increasing oxygen intake but leading to fast, shallow breathing
Immune system	Initial immune boost, followed by suppressed immune function with prolonged stress	Short-term stress increases **cytokine release** and neutrophil activity; chronic stress suppresses immune function via cortisol
Endocrine system	Elevated cortisol and adrenaline levels, altered thyroid function	**HPA axis activation** increases stress hormone secretion, affecting metabolism and hormonal balance
Gastrointestinal system	Nausea, stomach pain, diarrhea or constipation, changes in appetite	Cortisol and **autonomic nervous system** activation alters **digestive motility** and enzyme secretion
Integumentary system	Sweating, dry skin, acne breakouts, hair loss	Activation of sweat glands via **SNS**, altered skin oil production, and reduced blood flow to the skin
Reproductive system	Reduced libido, irregular menstrual cycles, erectile dysfunction	Chronic stress disrupts the balance of sex hormones (e.g., testosterone, estrogen), affecting reproductive health
Sleep-wake cycle	Insomnia, disrupted sleep patterns, fatigue	Dysregulation of **cortisol** and **melatonin** levels, impacting the ability to fall asleep and quality of rest
Cognitive function	Impaired memory, difficulty concentrating, decision-making challenges	Chronic elevated cortisol levels damage **hippocampal neurons**, leading to cognitive impairment and memory deficits

### Implications for health and disease

The dynamic interplay between stress and neutrophil function has profound implications for health and disease. Both acute and chronic stress can significantly influence the immune system, with implications ranging from enhanced pathogen defense to increased susceptibility to chronic diseases. Acute stress can temporarily boost immune responses, enhancing the body’s ability to combat infections^[43]^. During acute stress, elevated levels of stress hormones, such as adrenaline and cortisol, mobilize neutrophils from the bone marrow and increase their activity. This enhanced neutrophil response helps to quickly address infections and promote recovery. However, while acute stress can be beneficial in the short term, chronic stress has the opposite effect, leading to immune suppression and an increased risk of infections. Chronic exposure to stress hormones can impair neutrophil function, including reduced chemotaxis, phagocytosis, and ROS production^[44]^. This weakened immune response can make individuals more susceptible to common infections, such as respiratory and gastrointestinal illnesses. Chronic stress can contribute to the development and exacerbation of autoimmune diseases and chronic inflammatory conditions^[45]^. Prolonged stress results in sustained high levels of cortisol, which can disrupt the balance between pro-inflammatory and anti-inflammatory cytokines. This disruption can lead to chronic inflammation, a key feature of many autoimmune diseases. Conditions such as rheumatoid arthritis, lupus, and multiple sclerosis are characterized by inappropriate immune responses against the body’s own tissues. Stress-induced dysregulation of immune responses, including altered cytokine profiles and impaired neutrophil functions, can exacerbate these diseases and contribute to disease progression.

Chronic stress is a significant risk factor for cardiovascular diseases^[46]^. Persistent stress leads to chronic inflammation and elevated levels of pro-inflammatory cytokines, which can contribute to the development of atherosclerosis, hypertension, and heart disease. Neutrophils play a role in cardiovascular disease by contributing to inflammation and endothelial dysfunction. Stress-induced changes in neutrophil activity, such as increased ROS production and altered cytokine release, can accelerate the development of atherosclerotic plaques and increase the risk of cardiovascular events like heart attacks and strokes. Chronic stress is linked to metabolic disorders, including obesity, type 2 diabetes, and insulin resistance^[47]^. Stress-induced cortisol release promotes glucose production and insulin resistance, which can lead to metabolic dysregulation. Additionally, chronic stress can exacerbate inflammation, which plays a role in the development of metabolic syndrome. Neutrophils, through their inflammatory activities, can contribute to insulin resistance and the development of metabolic disorders. Elevated stress levels can lead to increased fat deposition and higher blood glucose levels, further complicating these conditions. Stress can influence cancer progression through effects on immune function and inflammation. Chronic stress can create a pro-tumorigenic environment by promoting chronic inflammation and enhancing the survival of cancer cells^[48]^. Neutrophils can both suppress and support tumor development, depending on the context. While they can kill cancer cells and produce anti-tumorigenic factors, chronic stress-induced neutrophil activity may also promote tumor growth by releasing growth factors, enzymes that degrade extracellular matrix, and inflammatory cytokines. The balance between these opposing effects can influence cancer progression and patient outcomes.

Neutrophils are essential for wound healing and tissue repair, but chronic stress can impair these processes. Acute stress can enhance neutrophil-mediated tissue repair through increased recruitment and activation^[49]^. However, chronic stress-induced neutrophil dysfunction can delay wound healing and impair tissue repair. Stress-related changes in neutrophil function, such as reduced phagocytic activity and increased apoptosis, can hinder the repair of damaged tissues and prolong recovery from injuries or surgeries. The impact of stress on mental health is well-documented, with chronic stress contributing to mood disorders such as depression and anxiety^[50]^. The relationship between stress, immune responses, and mental health is complex, with evidence suggesting that inflammation and immune dysregulation may play roles in these conditions. Chronic stress-induced changes in neutrophil activity and inflammatory cytokine levels can affect brain function and contribute to the development of mental health disorders. Stress can influence allergic reactions through its effects on the immune system^[51]^. While acute stress may enhance immune responses to allergens, chronic stress can shift the immune response toward a Th2-dominated profile, which is associated with increased allergic inflammation. Stress-induced alterations in neutrophil function and cytokine production can exacerbate allergic conditions such as asthma, allergic rhinitis, and eczema. Managing stress effectively is therefore important for controlling allergic reactions and improving quality of life for individuals with allergies.

Chronic stress has significant effects on gastrointestinal health, potentially leading to conditions such as irritable bowel syndrome (IBS) and inflammatory bowel diseases (IBD)^[52]^. Stress-induced changes in neutrophil function and cytokine release can contribute to gastrointestinal inflammation and dysregulation of gut microbiota. Stress can also exacerbate symptoms of existing gastrointestinal conditions, highlighting the need for stress management as part of a comprehensive approach to treating gastrointestinal diseases. Chronic stress may accelerate immune system aging, a phenomenon known as immunosenescence^[53]^. Prolonged stress can lead to persistent inflammation and immune system dysregulation, which are characteristic of aging. Stress-induced changes in neutrophil function, including increased inflammation and reduced response to infections, can contribute to the aging of the immune system. Addressing chronic stress through lifestyle changes and interventions can help mitigate the effects of immunosenescence and promote healthier aging.^[^54-58^]^

### Future research implications

The dynamic relationship between stress and immune health is a complex field, with significant potential for further exploration. While the current body of literature provides valuable insights into how stress affects immune function, many questions remain unanswered. Several key areas of research could help deepen our understanding of the mechanisms involved, refine therapeutic strategies, and improve patient outcomes.

1. **Elucidating the Molecular Mechanisms of Stress-Induced Immune Dysregulation**: Future studies should focus on identifying specific molecular pathways through which stress impacts immune cells, particularly neutrophils. Research is needed to unravel how chronic stress influences the expression of genes involved in neutrophil activation, apoptosis, and migration. Advanced techniques like single-cell RNA sequencing and proteomics can be employed to map stress-induced changes at the cellular level, offering a more precise understanding of how stress impairs immune function.^[59]^
2. **Investigating the Role of Stress on Immune Cell Subsets and Function**: While much research has focused on neutrophils and their immediate response to stress, the broader impact of stress on other immune cell subsets, such as T cells, B cells, and macrophages, remains underexplored. Studies should investigate how chronic stress modulates the balance between pro-inflammatory and anti-inflammatory immune responses, particularly in the context of autoimmune and chronic inflammatory diseases.^[60]^
3. **Longitudinal Studies on Chronic Stress and Immune Health**: The long-term effects of chronic stress on immune health are not fully understood. Longitudinal studies involving large, diverse populations are needed to examine how prolonged exposure to stress affects immune function over time. These studies should incorporate a wide range of stressors (e.g., psychological, physical, environmental) and consider how genetic and environmental factors influence individual susceptibility to stress-induced immune dysregulation.
4. **Exploring the Intersection of Neuroimmune and Psychoneuroendocrine Pathways**: The HPA axis and neuroimmune signaling pathways, such as the sympathetic nervous system (SNS), are critical mediators of the stress response. Future research should investigate how these systems interact to regulate immune function in the context of stress. Specifically, studies should focus on the role of stress hormones like cortisol and catecholamines in modulating immune cell function and inflammatory responses, with particular attention to their effects on neuroinflammation.
5. **Identifying Biomarkers for Stress-Induced Immune Dysfunction**: There is a need for reliable biomarkers to assess the impact of chronic stress on immune health. Identifying biomarkers, such as specific cytokines, immune cell profiles, or stress hormones, could help clinicians predict and monitor immune dysfunction in stressed individuals. Additionally, studying the role of stress-related epigenetic changes could provide valuable insights into how stress alters immune gene expression over time, contributing to immune dysregulation.
6. **Stress, Aging, and Immunosenescence**: Aging is associated with a gradual decline in immune function, known as immunosenescence. Research should explore how chronic stress accelerates this process, particularly in older adults. Understanding the interplay between stress and aging-related immune changes could provide important insights into the development of age-related diseases, such as cancer, cardiovascular disease, and neurodegenerative disorders. Studies focusing on interventions that mitigate stress-induced immunosenescence could help improve health outcomes in aging populations.
7. **Investigating the Impact of Stress on Vaccine Efficacy and Infectious Disease Outcomes**: The role of chronic stress in modulating vaccine responses and susceptibility to infections is a critical area for future research. Studies should assess how stress-induced immune suppression affects the ability to generate protective immune responses following vaccination. Additionally, research is needed to determine whether stress influences the severity of infectious diseases, particularly in vulnerable populations, such as those with underlying health conditions or immunocompromised individuals.
8. **Developing Stress-Reduction Interventions for Immune Health**: While stress-reduction techniques like mindfulness, yoga, and cognitive behavioral therapy (CBT) have been shown to improve psychological well-being, their effects on immune function are not fully understood. Future studies should evaluate the impact of these interventions on immune health, particularly in individuals experiencing chronic stress. Randomized controlled trials (RCTs) are needed to determine whether stress-reduction strategies can enhance immune function and reduce the risk of stress-related diseases.
9. **Personalized Medicine Approaches to Stress and Immune Dysfunction**: The individual variability in immune responses to stress highlights the need for personalized medicine approaches. Future research should explore how genetic, epigenetic, and environmental factors influence the immune system’s response to stress. This could lead to the development of personalized interventions tailored to an individual’s stress profile, potentially improving immune resilience and reducing the risk of immune-mediated diseases.
10. **Interdisciplinary Research Bridging Immunology, Psychology, and Neurology**: The future of stress and immune health research lies in interdisciplinary collaboration. Immunologists, psychologists, neurologists, and endocrinologists should work together to explore the complex interactions between stress, the immune system, and the CNS. Collaborative studies integrating different perspectives and methodologies will provide a more comprehensive understanding of the multifaceted relationship between stress and immune function, ultimately guiding the development of more effective interventions for stress-related diseases.

## Conclusion

Stress significantly influences neutrophil function and, by extension, affects various aspects of immune system health and disease. The complex mechanisms through which stress modulates neutrophil activity underscore the dual nature of stress effects – while acute stress can enhance immune responses and improve pathogen clearance, chronic stress generally leads to immunosuppression and exacerbates disease processes. **Acute Stress** triggers immediate physiological changes that can temporarily boost immune defenses. By increasing the mobilization and activity of neutrophils, acute stress enhances the body’s ability to respond to infections and manage injury. This beneficial effect, however, is short-lived and must be managed carefully to prevent transition to chronic stress conditions. **Chronic Stress**, on the other hand, leads to sustained high levels of stress hormones like cortisol, which can impair neutrophil function and contribute to a range of health issues. Chronic stress-induced neutrophil dysfunction results in increased susceptibility to infections, chronic inflammatory diseases, cardiovascular diseases, metabolic disorders, and even cancer. These effects are mediated through various mechanisms including hormonal regulation, neuroimmune interactions, altered cytokine profiles, and oxidative stress. **The Implications for Health and Disease** highlight the importance of stress management as a crucial component of maintaining overall health. Effective strategies for managing stress, such as cognitive-behavioral therapies, lifestyle modifications, and stress-reducing activities, are essential for preventing the negative impacts of chronic stress on the immune system. Additionally, understanding how stress affects neutrophil function can guide the development of targeted therapies for stress-related health conditions.

## Data Availability

Not applicable as this a review.

## Research registration unique identifying number (UIN)

Not applicable as this a review.
